# Beta-Blockers as Potential Adjuvants in Melanoma Treatment

**DOI:** 10.3390/toxics13110981

**Published:** 2025-11-14

**Authors:** Laura Rama, Mónica Almeida, Jiya Jose, Maria de Lourdes Pereira, Miguel Oliveira

**Affiliations:** 1 Centre for Environmental and Marine Studies (CESAM), Department of Biology, University of Aveiro, 3810-193 Aveiro, Portugal; monica.alm@ua.pt; 2 CICECO-Aveiro Institute of Materials, Department of Medical Sciences, University of Aveiro, 3810-193 Aveiro, Portugal; mlourdespereira@ua.pt; 3 Department of Biosciences, Rajagiri College of Social Sciences, Kochi 682039, Kerala, India; jiyajose@rajagiri.edu

**Keywords:** melanoma, drug repurposing, cancer cell lines, beta-blockers, combined exposures

## Abstract

Melanoma, in advanced stages, is the most invasive type of skin cancer, with currently available treatments showing limited efficiency. The number of melanoma cancer cases is expected to increase in the coming years, emphasizing the need for more efficient therapeutic strategies. The present study aimed to evaluate the potential of β-blockers, commonly used to treat cardiac conditions, to be repurposed for the treatment of melanoma. The effects of non-selective β-blockers (carvedilol and propranolol), β1 selective blockers (atenolol and metoprolol) and antineoplastics drugs (cisplatin and 5-fluorouracil) on the A375 melanoma cell line were studied, individually and in combined exposures, by assessing cell viability over a 72 h period. The 72 h half-maximal inhibitory concentrations (IC_50_s) determined for A375 cells allow the ranking of toxicity as: cisplatin (2.46 (1.87–3.38) μM) > 5-fluorouracil (4.77 (4.48–5.07) μM) > carvedilol (16.91 (15.47–18.99) μM) > propranolol (58.03 (57.08–59.11) μM) > atenolol and metoprolol (β1 selective blockers that exhibited no significant effect on the cell’s viability). The effects of combined exposures were also studied. Metoprolol and carvedilol exhibited synergistic interactions with cisplatin at specific concentrations. Overall, the data highlight the concentration-dependent nature of mixture effects and support the potential application of β-blockers melanoma treatment.

## 1. Introduction

Melanoma is one of the most aggressive forms of cancer. In 2022, more than 330,000 new cases and over 58,000 deaths were registered worldwide [1]. This disease has a 5-year survival rate of around 99% for early-stage localized tumors, which decreases with evolution of the disease to rates below 30% in the event of metastasis [2]. The number of melanoma cases is projected to exceed 470,000 by the year 2050, representing an increase in more than 40% [3]. The risk of developing melanoma can increase upon UV radiation exposure, either from direct sun exposure or tanning beds [4,5]. This malignancy arises from mutations in melanocytes, most commonly in the BRAF oncogene (V600E), which promotes abnormal and uncontrolled cellular growth [6].

Upon diagnosis, the most common treatment for melanoma, particularly in its early stages, is the surgical removal of the tumor, which is generally effective. However, advanced stages of the disease require additional therapeutic approaches. Radiotherapy is mainly used in metastatic cases or for palliative purposes to alleviate symptoms. Chemotherapy has shown low efficiency in melanoma and is mainly used in combination with other therapies, or as a last resort, since it has significant side effects on healthy cells. With medical and technological advances, new therapeutic strategies have emerged, such as targeted therapy, which offers a personalized approach, targeting the mutations responsible for cancer growth and progression, and immunotherapy which takes advantage of the immune system to diminish cancer progression. Even though both treatments are more efficient than chemotherapy and have less side effects, they are also more expensive and therefore inaccessible to some patients [7]. In certain cases, chemotherapy is the only viable option, highlighting the importance of optimizing treatment outcomes for all patients [8].

A new approach for cancer treatment improvement is drug repurposing/repositioning, which involves using already approved and clinically validated pharmaceuticals to treat different medical conditions. This approach is faster, more cost-effective, and safer than developing a new drug. The overall idea is to use these drugs, that may act as adjuvants in chemotherapy and increase treatment efficiency [9,10,11]. For example, drugs like triptolide (natural compound with anti-rheumatic, anti-bacterial, and anti-inflammatory properties) disulfiram (drug approved by the United States Food and Drug Administration (FDA) for alcoholism) and leflunomide (immunomodulatory drug used for arthritis treatment), have been proposed for drug repurposing to help in cancer treatment [12].

β-blockers are drugs that bind to the β-adrenergic receptors inhibiting their signaling pathways [13,14]. Commonly prescribed for patients with heart and vascular system conditions, these drugs are often taken at the same time as the chemotherapy treatment (i.e., in patients with heart conditions and cancer), with some studies reporting tumor mitigation and higher survival rates in those patients [15,16]. Since BRAF is the most common mutation found in cancer patients, BRAF inhibitors have been a targeted therapeutic approach with beneficial effects in many cases. However, the emergence of drug resistance to these inhibitors has become a major clinical challenge. Recent studies on copper ionophore disulfiram have demonstrated that, by targeting mitochondrial function, this compound can suppress melanoma with resistance to BRAF inhibitors [17]. β-blockers, particularly those targeting β_3_-adrenergic receptors have been shown to affect the tumor microenvironment (TME) and slow down melanoma progression [18]. There is evidence of the presence of many β-adrenergic receptors in cancer cells, of their important role in the proliferation of cancer cells, and regulation of cellular processes such as inflammation, angiogenesis, apoptosis, DNA damage repair, and cellular immune response that contribute to the initiation and progression of cancer [19]. Considering that melanoma is one of the cancers with more expression of β-adrenergic receptors, expressing the three classes: β1, β2, and β3 [20], the study of the effects of β-blockers appears highly relevant. The β-adrenergic signaling crosstalk with BRAF V600E mutation in melanoma is presented in Figure S3.

Recent systematic reviews and meta-analyses have provided valuable insights into the potential impact of β-blockers on cancer patients’ survival: how they may help slow down cancer progression [21], act as adjuvant agents in treatment and reduce the risk of recurrence [22], and their beneficial effect when combined with immunotherapy [23]. The projected rise in melanoma cases supports the urgency of enhancing treatment efficiency and overcoming therapeutic resistance [24]. In this context, β-blockers have emerged as promising candidates, although further scientific validation is still required [25]. Therefore, this work takes an in vitro approach to better understand the effect of β-blockers on melanoma cells alone, and combined with antineoplastic drugs, to evaluate its potential to increase the efficiency of the available treatments for this type of cancer. The effects of combined exposures were compared to the individual effects and analyzed.

## 2. Materials and Methods

### 2.1. Chemicals

Atenolol (CAS: 29122-68-7), metoprolol (CAS: 56392-17-7), carvedilol (CAS: 72956-09-3), propranolol (CAS: 318-98-9), 5-fluorouracil (CAS: 51-21-8), cisplatin (cis-Diammineplatinum (II) Dichloride; CAS: 15663-27-1), and MTT (thiazolyl blue tetrazolium bromide; CAS: 298-93-1) were obtained from TCI Chemicals (Antwerp, Belgium). All other chemicals were analytical grade from Sigma-Aldrich (Madrid, Spain). Stock solutions of atenolol, metoprolol, propranolol, and 5-fluorouracil were prepared in ultra-pure water whereas carvedilol and cisplatin were prepared in dimethyl sulfoxide (DMSO). The stock solutions were at a concentration of 50 mM, except 5-fluorouracil which was at 20 mM. The highest concentration of DMSO in cell viability assay were 0.2% for carvedilol and 0.5% for cisplatin.

### 2.2. Biological Model

The A375 cells were kindly provided by Dr. João Ramalho from the Netherlands Cancer Institute in Amsterdam, Netherlands. These cells originated from a primary malignant melanoma that exhibited an epithelial morphology. Cultures were maintained in Dulbecco’s Modified Eagle Medium (DMEM) supplemented with 2 mM L-Glutamine, 10% Fetal Bovine Serum (FBS), 100 U/mL Penicillin G, 100 μg/mL Streptomycin, and 2.5 μg/mL of Amphotericin B (Biowest, Nuaillé, France). Cells were routinely maintained in 10 cm diameter culture dishes (Orange Scientific, Braine-l’Alleud, Belgium), in a humified cell incubator, at 37 °C degrees, supplemented with 5% CO_2_. All assays were performed in flat-bottom clear 96-well plates suitable for colorimetric measurements.

### 2.3. Experimental Design

#### 2.3.1. Individual Exposures

Cells were seeded in 96-well plates at a density of 10,000 cells per well and allowed to adhere overnight. The following concentration ranges of selected pharmaceuticals were tested: atenolol and metoprolol (3.90625, 7.8125, 15.625, 31.25, 62.5, 125, 250, 500 μM); carvedilol (0.78125, 1.5625, 3.125, 6.25, 12.5, 25, 50, 100 μM); propranolol (10, 25, 50, 100, 125, 150, 200, 250 μM); cisplatin (0.0032, 0.016, 0.08, 0.4, 2, 10, 50, 250 μM); and 5-fluorouracil (1.5625, 3.125, 6.25, 12.5, 25, 50, 100, 200 μM). Cell viability was assessed every 24 h up to 72 h. The working solutions were prepared by serial dilution of the stocks in DMEM. Each assay had 3 technical replicas, and a minimum of 3 independent assays were performed. Solvent controls were also performed assessing the effect of the highest concentrations of DMSO used in the tests.

#### 2.3.2. Combined Exposures

Combined exposure assays were conducted following the same procedure as for individual treatments but testing binary mixtures of the most promising compounds. Combined exposures to carvedilol and propranolol; cisplatin and metoprolol; cisplatin and carvedilol; and cisplatin and propranolol were performed. The concentrations were selected based on a factorial design, using the 48 h IC_50_ value determined in the individual exposure assays as one toxic unit (TU). Fractions of this value (0.25; 0.5; 0.75; and 1 (TU)) were tested for each compound. For metoprolol, since an IC_50_ could not be established within the tested concentrations range, the highest concentration used in the individual exposures was selected for the combined exposures. Cell viability was assessed after 48 h. Each experimental condition had 3 technical replicas, and 3 independent assays were performed.

### 2.4. Cell Viability Assay

Cell viability was assessed based on the cellular metabolic activity through the 3-(4,5-dimethylthiazol-2-yl)-2,5-diphenyltetrazolium bromide (MTT) assay. Measurements were performed at 3 different time points for the individual exposures (24, 48, and 72 h) and at a single time point (48 h) for the combined exposures. MTT was diluted in phosphate-buffered saline (PBS) (0.5 mg/mL) and added to the 96-well plate. After one hour of incubation, the medium was removed, and the formazan crystals formed by cell metabolism were dissolved with DMSO. The absorbance of the resulting solution was read at 570 and 690 nm (baseline measure) [26], using a microplate spectrophotometer (Multiskan Spectrum–Thermo Scientific, Waltham, MA, USA).

### 2.5. Data Analysis

Cell viability was calculated as the percentage of control, following Riss (2016) [26]. The results from the viability assays were presented as the means of the 3 independent assays. Data were fitted into a model of a nonlinear regression with variable slope (four parameters) using GraphPad 9, and inhibitory concentrations (IC_10_, IC_25_ and IC_50_) were calculated. A two-way ANOVA followed by Tukey’s post hoc test were also performed, with time and concentration set as factors, with *p* < 0.05 set as significance value.

In the binary combinations, the interaction between the pharmaceuticals were studied through Synergy Finder 3.0 (SF) [27], an online tool commonly used in cancer research studies. Two distinct models were applied: Loewe additivity model for drugs sharing similar mode of action, such as both β-blockers, and Bliss independence model for drugs with different mechanisms, such as β-blockers and antineoplastic agents. Within Synergy Finder 3.0, interactions strength is expressed as overall delta (δ) score. Scores support synergy when the delta value is above 10; between 10 and 5 are not significant but reveal a tendency for synergism. Between 5 and −5, shows additive effects; between −5 and −10 shows a tendency for antagonism and below −10 shows antagonism. We normalized results (viability as percentage of control) and used the default parameters, including log10 transformation of concentration.

## 3. Results

### 3.1. Individual Exposure

#### 3.1.1. β-Blockers

A375 cells were exposed up to 72 h to the β1 blockers, atenolol and metoprolol, at concentrations up to 500 μM. No significant effects on cell viability were observed within the concentration range tested. The statistical analysis confirmed that concentration and time point had no significant influence on cell viability (Figure S1; Table S1).

**Figure 1:**
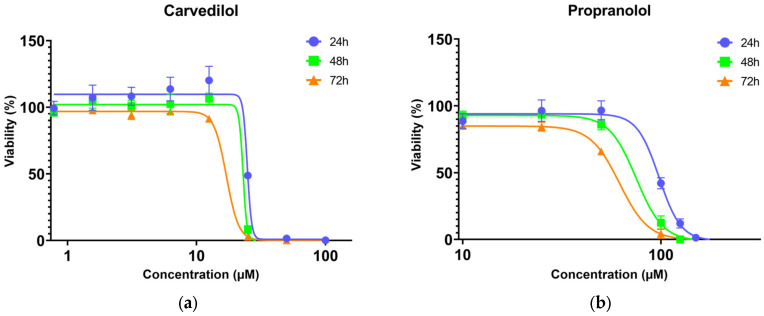
Dose–response nonlinear regression curves (four-parameter logistic curves) of A375 cells viability (MTT assay) after 24, 48, and 72 h exposure to (**a**) carvedilol and (**b**) propranolol. Data points correspond to mean viabilities ± standard error.

Concerning non-selective β blockers, concentrations up to 100 μM for carvedilol and 250 μM for propranolol were tested. After 24 h exposure, cell viability was completely inhibited by carvedilol concentrations equal or higher than 50 μM whereas a similar effect was observed for propranolol concentrations equal or higher than 150 μM. The A375 cells viability data dose–response curves (four-parameter logistic curves), presented in Figure 1, show a decrease in cell viability with exposure duration, as reflected by the decrease in ICs values from 24 to 72 h (Table 1). The 72 h IC_50_ values of 16.91 (15.47–18.99) μM for carvedilol and 58.03 (57.08–59.11) μM for propranolol show that carvedilol is more cytotoxic to A375 cells (Table 1 and Table S4). The two-way ANOVA reveals that both time and concentration significantly affect the cytotoxicity of carvedilol (*p* < 0.05), with effects increasing over time. Similar results were obtained for propranolol, although with more pronounced differences in cell viability between time points (*p* < 0.0001) (Table S2).

#### 3.1.2. Antineoplastic Drugs

**Figure 2:**
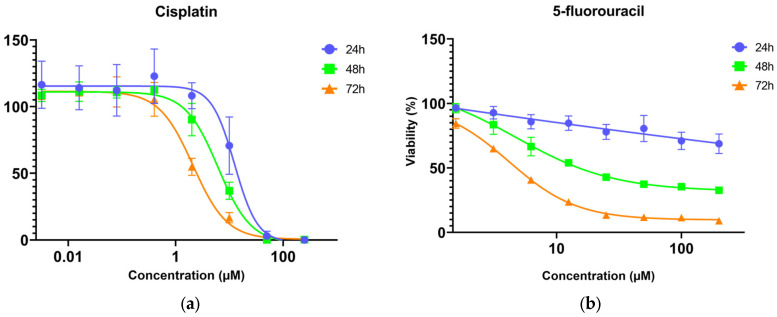
Dose–response nonlinear regression curves (four-parameter logistic curves) of A375 cells viability (MTT assay) after 24, 48, and 72 h exposure to (**a**) cisplatin and (**b**) 5-fluorouracil. Data points correspond to mean viabilities ± standard error.

The cell viability data show that cisplatin was more toxic to A375 cells than 5-fluorouracil, with both drugs showing time-dependent effects. The cytotoxicity of cisplatin increased significantly with exposure duration (*p* < 0.05). Nevertheless, at cisplatin concentrations higher than 50 μM, cell viability reached 0% across all time points (Figure 2). After 24 h exposure, 5-fluorouracil had a slight effect on the viability, but cytotoxicity increased with time (*p* < 0.0001). After 72 h, cell viability decreased to below 20% at 25 μM and higher concentrations but never reached 0%. The estimated 72 h IC_50_ values were 2.46 (1.87–3.38) μM for cisplatin and 4.77 (4.48–5.07) μM for 5-fluorouracil (Table 1 and Table S4). The two-way ANOVA revealed that concentration also had a significant impact on the cytotoxicity of cisplatin (*p* < 0.05) and 5-fluorouracil (*p* < 0.0001) (Table S3).

### 3.2. Combined Exposures

**Figure 3:**
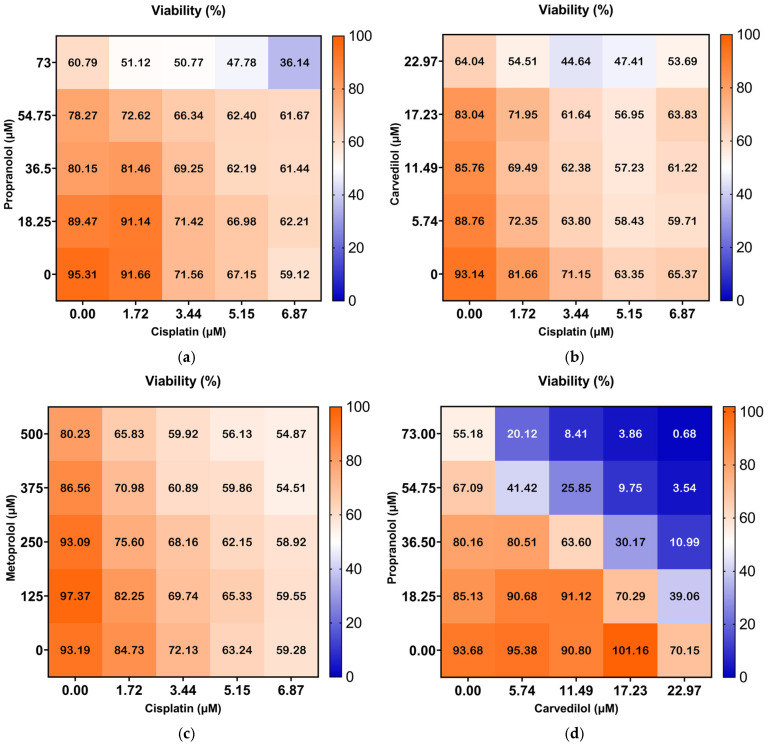
Heatmaps of A375 cell viability (expressed as percentage of control) after 48 h exposure to the binary mixtures of (**a**) cisplatin and propranolol, (**b**) cisplatin and carvedilol, (**c**) cisplatin and metoprolol, and (**d**) carvedilol and propranolol.

Following the individual assays, the antineoplastic cisplatin was selected for the combined exposures, based on its individual effects (the most promising antineoplastic) and the beta-blockers metoprolol, carvedilol, and propranolol. They were tested in combined exposures, following a factorial design, based on the 48 h IC_50_ determined for each drug. Cell viability was assessed after 48 h of combined exposure. The results are presented as a heatmap (Figure 3 and Figure S2).

**Figure 4:**
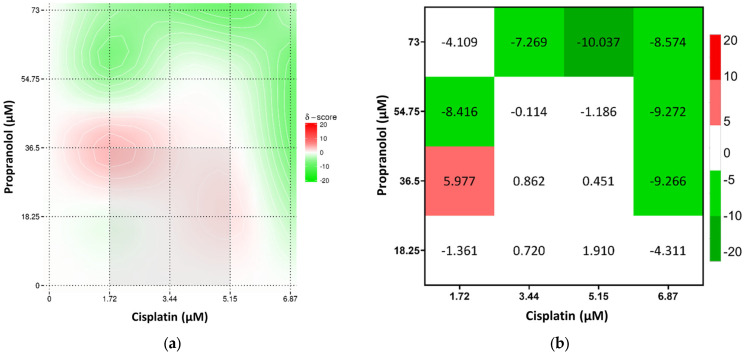
Results of drug interaction after 48 h of exposure to cisplatin and propranolol, analyzed using the Bliss model. (**a**) Synergy map representing interaction intensity, and (**b**) a combination index matrix showing the interaction profile across concentration ranges.

**Figure 5:**
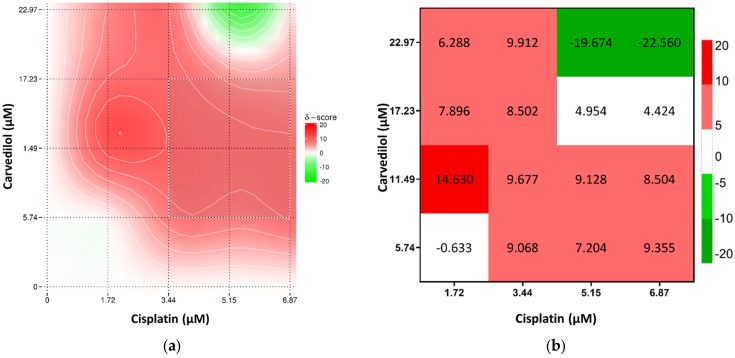
Results of drug interaction after 48 h of exposure to cisplatin and carvedilol, analyzed using the Bliss model. (**a**) Synergy map representing interaction intensity, and (**b**) a combination index matrix showing the interaction profile across concentration ranges.

**Figure 6:**
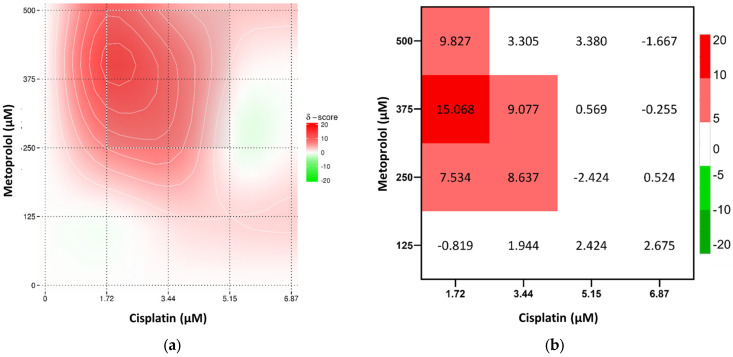
Results of drug interaction after 48 h of exposure to cisplatin and metoprolol, according to the Bliss model. (**a**) Synergy map representing interaction intensity, and (**b**) a combination index matrix showing the interaction profile across concentration ranges.

**Figure 7:**
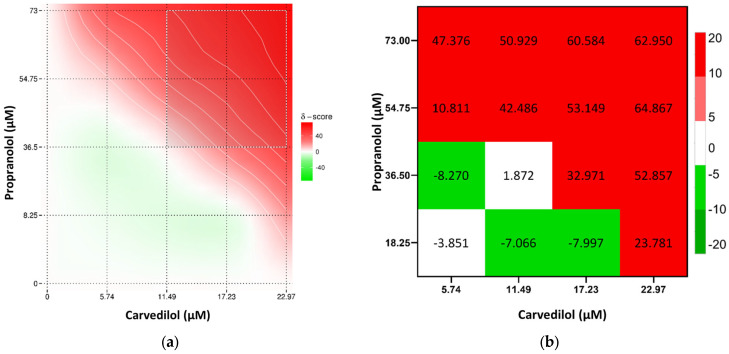
Results of drug interaction after 48 h of exposure to carvedilol and propranolol, analyzed using the Loewe model. (**a**) Synergy map representing interaction intensity, and (**b**) a combination index matrix showing the interaction profile across concentration ranges.

Overall, the combination of cisplatin with propranolol showed predominantly additive effects. However, some concentrations displayed antagonistic tendencies, especially at 5.15 μM of cisplatin and 73 μM of propranolol. A slight synergistic interaction was observed at 1.72 μM cisplatin and 36.5 μM of propranolol but too low to be considered synergism (Figure 4). Cisplatin and carvedilol displayed more synergistic tendencies, with a synergy peak at 1.72 μM of cisplatin and 11.49 μM of carvedilol. However, at higher concentrations of both drugs (5.15 μM and 6.87 μM of cisplatin with 22.97 μM of carvedilol) antagonism was observed (Figure 5). Even though metoprolol alone did not have a significant effect on A375 cells, when combined with cisplatin, it was able to increase the drug effect, with synergistic interaction observed at 1.72 μM cisplatin and 375 μM of metoprolol, and some synergistic tendency at low cisplatin concentrations (Figure 6). The potential of combining the two most effective β-blockers, carvedilol and propranolol, was also tested. The results showed that higher concentrations of both drugs had a high synergistic effect, whereas low concentrations exhibited antagonistic tendency (Figure 7b).

Overall, the combinations of carvedilol and metoprolol with cisplatin were the most promising, increasing the cisplatin cytotoxic action (Table 2). Moreover, the β-blockers combination exhibited high synergistic action.

## 4. Discussion

Drug repurposing has been proposed as a viable approach to improve cancer treatment [12]. Some drugs, such as some β-blockers, commonly prescribed for heart conditions have been suggested to help mitigate the cancer progression [15]. Data of the present study support the potential of some β-blockers like carvedilol and propranolol for treatment of melanoma, as both drugs induced decreases in A375 cells’ viability in the individual exposures. The cytotoxicity of carvedilol toward melanoma cell lines has been previously reported. This drug showed a higher toxicity towards cells like C32 and A2058, with a 72 h IC_50_ of 8.07 μM and 5.58 μM [28], compared to A375 cells (72 h IC_50_ obtained in the present study of 16.91 μM). However, carvedilol exerted lower toxicity toward Fem-x melanoma cell lines with a 48 h IC_50_ of 32.17 μM [29], compared to 22.97 μM obtained in this study. Carvedilol has also shown potential to mitigate other types of cancer (e.g., breast cancer) both in in vitro and in vivo studies [30,31], and potential as co-adjuvant in radiotherapy against uveal melanoma [32]. Moreover, long-term use of carvedilol has been associated with a decreased risk of gastrointestinal and lung cancer [33]. Propranolol has also been tested in different studies aiming to assess its potential use for the treatment of different cancers. Overall, decreased proliferation of the cells, induction of apoptosis, and overall prevention of cancer progression have been reported. These effects were reported for breast [34], liver [35], prostate [36], lung [37], and gastric [38] cancer cell lines. In the present study, propranolol exhibited cytotoxic effects on A375 cells, with IC_50_ values of 58.03 μM and 96.04 μM after 24 h and 72 h exposure, respectively. These results are in line with the findings of Zhou et al. (2016) that reported IC_50_ values of 65.33 μM and 98.17 μM for the same cell line and exposure periods [39], and with Wrobel et al. (2015) findings that reported mortality higher than 80% for A375 cells after a 72 h exposure to 100 μM propranolol [40]. However, the data from the present study revealed that other types of β-blockers, like atenolol and metoprolol, do not significantly affect A375 cell viability when tested individually. Despite the lack of effects on A375 cell viability observed in this study, atenolol has been reported to exert toxicity to colorectal cancer cells (HT-29) with a 24 h IC_50_ of 52.9 μM [41] and able to help decrease angiogenesis and tumor growth in breast cancer, when combined with metformin in xenografts of nude mice [42]. For metoprolol, a single study reported cytotoxicity in leukemic T cell (MOLT-4) and monocyte (U937) cell lines, but only at very high concentrations (>1000 μM) [43]. This drug has also been associated with cardioprotective effects on healthy cells against chemotherapy [44,45].

Overall, data from the individual exposure show that the carvedilol is the most toxic β-blocker to A375 cells, followed by propranolol, whereas β1-selective blockers atenolol and metoprolol showed no significant toxicity within the tested concentration range. The lower toxicity of these selective β-blockers compared to the non-selective ones may be associated with lower expression of β1 receptors than β2 receptors in A375 cells [46]. However, the limited effects upon individual exposure do not necessarily indicate lack of value in cancer treatment when combined with an antineoplastic agent.

Cisplatin is one of the most widely used chemotherapeutic agents for various cancers including malignant melanoma [47], showing relevant efficiency. The results of the present study showed that A375 cells are very sensitive to this compound, as demonstrated by the 72 h IC_50_ of 2.46 μM, which is approximately half of the IC obtained for the other tested antineoplastic drug, 5-fluorouracil (72 h IC_50_ of 4.77 μM). Other in vitro studies have also demonstrated high toxicity of this drug towards melanoma cells. A 72 h IC_50_ of 0.5 μg cisplatin/mL (1.66 μM) was reported for B16F10, a murine melanoma cell line [48]. 5-fluorouracil is used in the treatment of many types of cancer such as colorectal cancer [49], gastric cancer [50], head and neck squamous cell carcinoma (HNSCC) [51], and skin cancer [52]. In the present study, A375 cells’ exposure to 5-fluorouracil elicited a time-dependent effect, with cells maintaining a high viability at 24 h, which slowly decreased at 48 and 72 h. This effect may be associated with 5-fluorouracil mode of action that acts on cells at the S phase of the cell cycle, making the effect noticeable once the cell starts mitosis. The analysis of the available literature reveals that the reported ICs for this drug vary considerably, even within the same type of cancer. For colorectal cancer cells HCT116 and HT29, the 72 h IC_50_ of 5-fluorouracil were estimated as 13.72 μM and 106.8 μM, respectively, whereas for HCM myocytes, the 72 h IC_50_ was 4.866 μM [53]. The observed differences likely reflect variations in cell replication rates and sensitivity to the drug.

In this study, the effects of β-blockers in combined exposures were assessed after 48 h, a time frame which ensures at least one cell cycle, while minimizing drug degradation and nutrient depletion, which can impact cell culture conditions. This approach provides a reliable window of stable conditions to assess drug interactions. Although some of the concentrations tested may not be directly translatable to clinical settings, they allow the exploration of potential off-target or repurposing effects beyond conventional use. In addition, β-blockers have a short biological half-time and are cleared from systemic circulation after 48 h.

Data from the individual exposures revealed a high potential of cisplatin and propranolol to be used in the treatment of melanoma cancers. Surprisingly, the combined exposure to cisplatin and propranolol revealed an antagonistic effect, with propranolol decreasing cisplatin toxicity. These results are not in agreement with data from other studies assessing the combined effects of these two drugs. A study with PC9 and A549 (human lung adenocarcinoma cells) found that when exposed to propranolol (1–50 μM) beforehand, cells were more sensitive to the cisplatin treatment (0.2–1 μM) [54]. However, there is a clear experimental design difference between the two studies: the later study used a sequential combination which appeared to have made cells more sensitive while in the present study a simultaneous exposure was performed. A study addressing the combined effects of propranolol and the chemotherapy drugs 5-fluorouracil and paclitaxel in various types of cell lines (breast adenocarcinoma (MCF-7, MDA-MB-231 and SKBR3); breast epithelial (HBL-100); neuroblastoma (SK-N-SH); non-small cell lung carcinoma (A549); glioblastoma (U87); and vascular endothelial (HMEC-1 and BMH29L)) cells lines revealed that the outcomes varied depending on cell type and specific cellular characteristics, with synergistic, antagonistic, or additive effects reported [55].

The combination of cisplatin with carvedilol elicited synergistic effects, especially when combined at 1.72 μM of cisplatin and 11.49 μM of carvedilol. Previous studies have shown that carvedilol helps prevent cisplatin-induced apoptosis in healthy renal tubular epithelial cells [56] through its antioxidant properties that can inhibit the activation of caspase 9, a protein necessary to the pathway leading to apoptosis, and a mechanism associated with cisplatin-induced nephrotoxicity [56]. For the combination metoprolol and cisplatin, higher synergism was observed at concentrations of 1.72 μM cisplatin and 375 μM of metoprolol. Thus, although metoprolol alone had no significant effect on A375 cells, it markedly enhanced cisplatin’s cytotoxicity when combined.

Overall, based on the analysis of the drug interactions, both carvedilol and metoprolol appear to be the most promising β-blockers for melanoma treatment. These drugs paired with low concentrations of cisplatin revealed a synergic effect and more impactful treatment action, making them possible adjuvants in chemotherapy against melanoma.

Despite the relevant findings of the present study, the mechanisms involved in the observed responses must be explored in following studies. In vitro studies often employ a shorter exposure period (e.g., 24 h) to assess acute effects, but extending to 48 h provides greater relevance to real-world pharmacokinetic scenarios, particularly for chronic or repeated dosing regimens. This exposure duration is considered to allow the study of phenomena such as drug accumulation, metabolite formation, enzyme induction or inhibition, and delayed cellular responses that may not manifest in shorter assays [57]. The translation of in vitro to in vivo is a major challenge that will have to be addressed in future studies. In vitro doses, often derived from cell-based assays (e.g., EC_50_ for efficacy or IC_50_ for toxicity), are typically higher and not directly comparable to in vivo levels due to the differences in terms of absorption, distribution, metabolism, excretion processes, protein binding, and tissue distribution but provide relevant information that must take into consideration safety margins (in vitro doses are often 10–100 fold higher than in vivo-equivalent administered doses). Future studies should also consider the mode of actions involved in drug interaction. For example, the study of key proteins (e.g., p-ERK, caspase-3, and Bcl-2 family proteins) may allow a better understanding of β-blockers’ effects on signaling pathways to cisplatin response. The quantification of reactive oxygen species production can provide information on the potential role of carvedilol’s antioxidant properties on the observed effects. CRISPR could be used to do a knockdown of β2-adrenergic receptors to confirm their role in the signaling pathway. While direct molecular confirmation was beyond the scope of the present study, the obtained data emphasize future studies to address these targets. While this study findings highlight the promising adjuvant role of β-blockers, such as carvedilol, some considerations must be taken into account. A relevant limitation of this study in the context of melanoma research is that the studied cells, A375, have the homozygous BRAF V600E mutation. This model effectively captures constitutive MAPK/ERK hyperactivation but may not fully represent the genetic heterogeneity observed in patient cohorts, where BRAF V600E often coexists with heterozygous mutations, additional oncogenic drivers (e.g., NRAS or PTEN alterations), or variable tumor subclones. Such heterogeneity can influence adrenergic signaling responses and therapeutic outcomes, potentially limiting the generalizability of the present in vitro observations to diverse clinical scenarios. Future studies should incorporate patient-derived organoids or cell lines with mixed mutational profiles to better mimic real-world tumor dynamics. Although carvedilol’s antioxidant effects appear beneficial in the TME, in vivo applications must consider potential off-target cardiac effects. As a non-selective β-blocker with alpha-1 antagonism, carvedilol can induce bradycardia, hypotension, or exacerbation of heart failure in susceptible individuals, particularly at doses overlapping with therapeutic windows (e.g., unbound concentrations of 0.2–2 nM). Careful dose optimization and monitoring in clinical trials should be considered at more advanced stages to mitigate potential cardiovascular adverse events [58]. Thus, these findings may not apply to heterogenetic tumors. By following only an in vitro approach, cardiac off-target effects were not assessed; however recent studies correlated propranolol activation to antitumoral immunity through T-cell activation [59]. Xerographs or other in vivo models would be a necessary step before progressing to clinical trials, to assess pharmacokinetic, pharmacodynamic, and TME interactions. Despite the promising in vivo data, the clinical trials mostly use propranolol, some of which have been cancelled [60].

## 5. Conclusions

Overall, selective β1-blockers did not significantly affect A375 cells viability in individual exposures, unlike non-selective β-blockers. Carvedilol was the β-blocker that was most toxic to the melanoma cells while cisplatin was the most toxic antineoplastic drug. Based on the 72 h cell viability data, the toxicity of the tested drugs to the A375 cells can be ranked as cisplatin > 5-fluouracil > carvedilol > propranolol.

Data from the combined exposures scenarios support the potential of carvedilol and metoprolol for cancer treatment. These drugs appear to exert dose-dependent effects capable of enhancing cisplatin cytotoxicity at lower concentrations through synergistic interactions. In contrast, propranolol demonstrated an opposite trend at higher concentrations, seemingly protecting cells from cisplatin-induced cytotoxicity.

Future studies should consider ternary combinations, involving both carvedilol, propranolol, and cisplatin. Additionally, future studies should consider testing other types of pharmaceuticals that can be taken while undergoing chemotherapy (e.g., antidepressants, diabetes medication) to assess its impact on treatment efficiency. The effects of β3 selective blockers should also be studied as a considerable part of the adrenergic receptors expressed in melanoma are β3 [61]. Despite the relevance of these study findings, these conditions should also be replicated on normal skin cell lines, to validate beta-blockers as potential adjuvants in chemotherapy.

## Figures and Tables

**Table 1:** Half-maximal inhibitory concentrations (IC_50_) of carvedilol, propranolol cisplatin, and 5-fluorouracil for A375 cells, after 24, 48, and 72 h of exposure. ICs were calculated through interpolation of the MTT assay viability data, using a nonlinear regression, and are expressed as μM. NC denotes that the ICs obtained were out of the concentrations range tested.

	IC_50_ (μM)		
	24 h	48 h	72 h
Carvedilol	24.95 (13.06–47.26)	22.97 (13.24–39.39)	16.91 (15.47–18.99)
Propranolol	96.04 (87.36–100.81)	73.00 (68.67–77.05)	58.03 (57.08–59.11)
Cisplatin	14.01 (11.04–23.08)	6.87 (5.58–8.48)	2.46 (1.87–3.38)
5-fluorouracil	NC	15.10 (13.76–16.69)	4.77 (4.48–5.07)

**Table 2:** Summary of interactions registered following Synergy Finder 3.0 analysis.

Drug Combination	Overall δ Score	Description	Peak Synergy Concentrations
Cisplatin + Propranolol	−3.028 ± 5.43	Additive interaction with some antagonistic tendencies observed at higher concentrations of both drugs	No synergy peak
Cisplatin + Carvedilol	5.949 ± 5.97	Additive interaction with synergistic tendencies at lower concentrations of both drugs and antagonism at higher concentrations.	1.72 μM of cisplatin and 11.49 μM of carvedilol
Cisplatin + Metoprolol	4.098 ± 5.46	Additive interaction with synergistic tendencies at lower concentrations of cisplatin	1.72 μM of cisplatin and 375 μM of metoprolol
Propranolol + Carvedilol	27.633 ± 7.45	Synergistic interaction at higher concentrations of both drugs, with some antagonistic tendencies at lower concentrations.	Most of high concentrations combinations

## Data Availability

The data presented in this study are available on request from the corresponding authors.

## Supplementary Materials

The following supporting information can be downloaded at: https://www.mdpi.com/article/10.3390/toxics13110981/s1, Figure S1: Cell viability after exposure to atenolol and metoprolol; Figure S2: Heatmaps from Synergy Finder as percentage of cell metabolism inhibition for cisplatin and propranolol (a), cisplatin and carvedilol (b), cisplatin and metoprolol (c) and propranolol and carvedilol (d). Figure S3: β-adrenergic signaling crosstalk with BRAF V600E mutation in melanoma. Table S1: Statistical results of a two-way ANOVA for atenolol and metoprolol exposure; Table S2: Statistical results of a two-way ANOVA for carvedilol and propranolol; Table S3: Statistical results of a two-way ANOVA for cisplatin and 5-fluorouracil; Table S4: Inhibition concentrations (IC_10_ and IC_25_) of carvedilol, propranolol cisplatin and 5-fluorouracil.
