# Supplementary material for: Beta-Blockers as Potential Adjuvants in Melanoma Treatment
Source: Toxics. 2025 Nov 14;13(11):981. doi: 10.3390/toxics13110981 (PMC12656413; doi:10.3390/toxics13110981)
Supplement: Supplementary file 1 [file toxics-13-00981-s001.zip › toxics-3952450-supplementary.pdf]

Supplementary materials

Beta-blockers as potential adjuvants in melanoma treatment

Laura Rama, Mónica Almeida, Jiya Jose, Maria L. Pereira and Miguel Oliveira

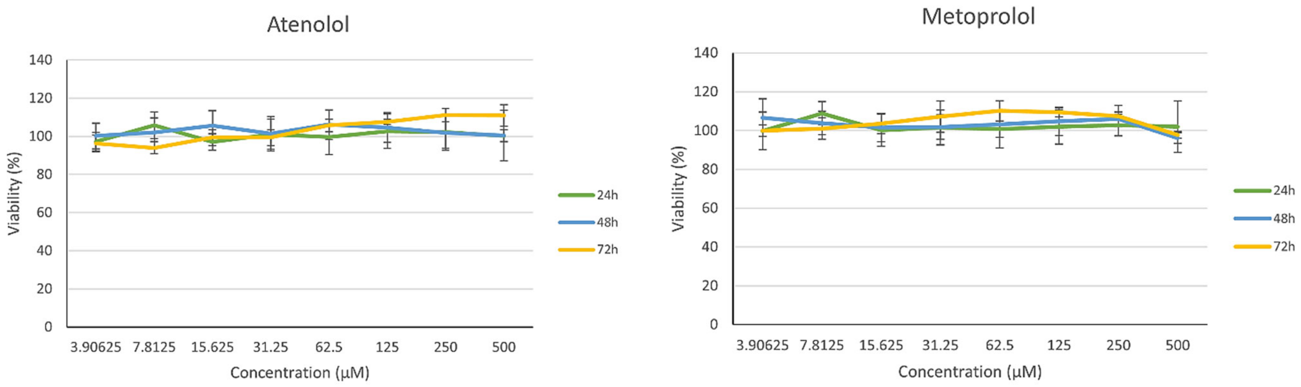

(a) (b)

Figure S1. Cell viability of A375 after exposure to different concentrations of atenolol (a) and metoprolol (b) at 3 timepoints: 24, 48 and 72h.

Table S1. Effect of concentration and time on the viability of A375 cells exposed to β1 selective, atenolol and metoprolol: Statistical results of a two-way ANOVA ($p<0.05$). [F-value (DFn; DFd) and p-value].

**Atenolol**		
Concentration x Time	F (14; 32) = 1.117	p=0.3812
Concentration	F (7; 16) = 0.1839	p=0.9849
Time	F (2; 32) = 0.7435	p=0.4835
**Metoprolol**		
Concentration x Time	F (14; 32) = 0.6899	p=0.7671
Concentration	F (7; 16) = 0.1432	p=0.9927
Time	F (2; 32) = 0.6254	p=0.5414

Table S2 Effect of concentration and time on the viability of A375 cells exposed to non-selective, carvedilol and propranolol: Statistical results of a two-way ANOVA ($p<0.05$). [F-value (DFn; DFd) and p-value].

**Carvedilol**		
Concentration x Time	F (20; 32) = 2.909	P=0.0034
Concentration	F (10; 16) = 224.6	P<0.0001
Time	F (2; 32) = 10.54	P=0.0003
**Propranolol**		
Concentration x Time	F (20; 32) = 6.030	P<0.0001
Concentration	F (10; 16) = 256.5	P<0.0001
Time	F (2; 32) = 17.40	P<0.0001

Table S3. Effect of concentration and time on the viability of A375 cells exposed to antineoplastics, cisplatin and 5-fluorouracil: Statistical results of a two-way ANOVA ($p<0.05$). [F-value (DFn; DFd) and p-value].

**Cisplatin**		
Concentration x Time	F (20; 32) = 1.363	P=0.2120
Concentration	F (10; 16) = 31.31	P<0.0001
Time	F (2; 32) = 3.845	P=0.0319
**5-fluorouracil**		
Concentration x Time	F (20; 32) = 6.658	P<0.0001
Concentration	F (10; 16) = 43.54	P<0.0001
Time	F (2; 32) = 117.2	P<0.0001

Table S4. Inhibition concentrations (IC_{10} and IC_{25}) of carvedilol, propranolol cisplatin and 5-fluorouracil for A375 cells, after 24, 48 and 72 hours exposure. ICs were calculated through interpolation of the MTT assay viability data, using a nonlinear regression, and are expressed as μM. NC denotes that the ICs obtained were out of the concentrations range tested.

Drug	IC	24h	48h	72h
Carvedilol	IC_{10}	23.37 (12.50 - 46.17)	21.36 (12.57 - 38.49)	12.84 (11.87 - 14.85)
	IC_{25}	24.06 (12.78 - 46.50)	22.12 (12.94 - 38.77)	14.88 (13.82 - 16.91)
Propranolol	IC_{10}	65.78 (NC - 83.31)	43.69 (31.91 - 50.21)	NC
	IC_{25}	82.04 (71.14 - 90.81)	59.85 (55.71 - 64.76)	43.88 (42.90 - 44.81)
Cisplatin	IC_{10}	6.66 (3.95 – 8.94)	2.19 (1.54 – 3.02)	0.75 (0.45 – 1.12)
	IC_{25}	9.12 (6.27– 11.96)	3.62 (2.75 – 4.58)	1.25 (0.86 – 1.68)
5-fluorouracil	IC_{10}	4.40 (NC – 9.63)	2.25 (2.06 – 2.43)	1.13 (NC)
	IC_{25}	63.49 (27.66 – NC)	4.47 (4.14 – 4.82)	2.29 (2.12 – 2.46)

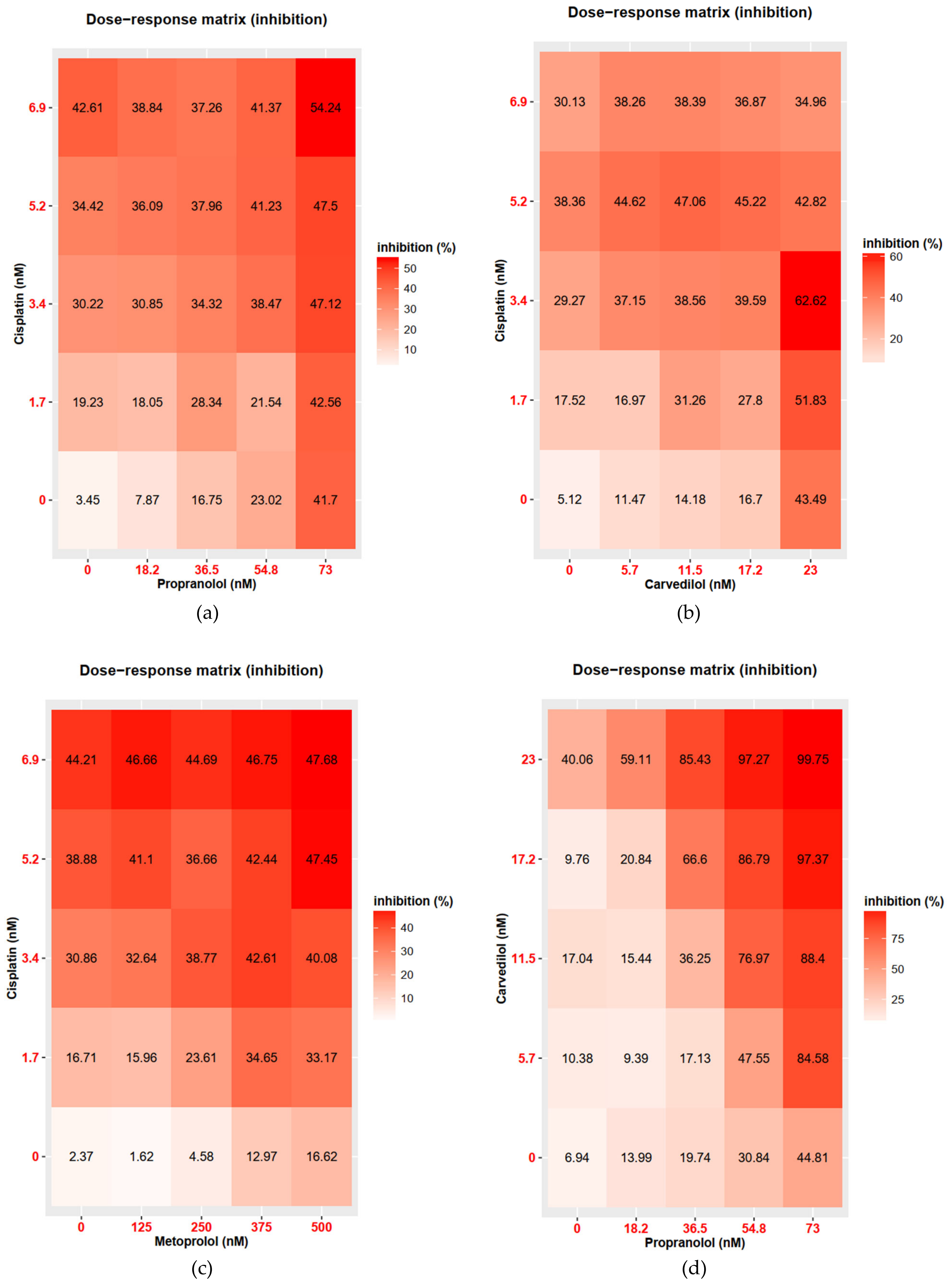

Figure S2: Heatmaps from Synergy Finder as percentage of cell metabolism inhibition for cisplatin and propranolol (a), cisplatin and carvedilol (b), cisplatin and metoprolol (c) and propranolol and carvedilol (d).

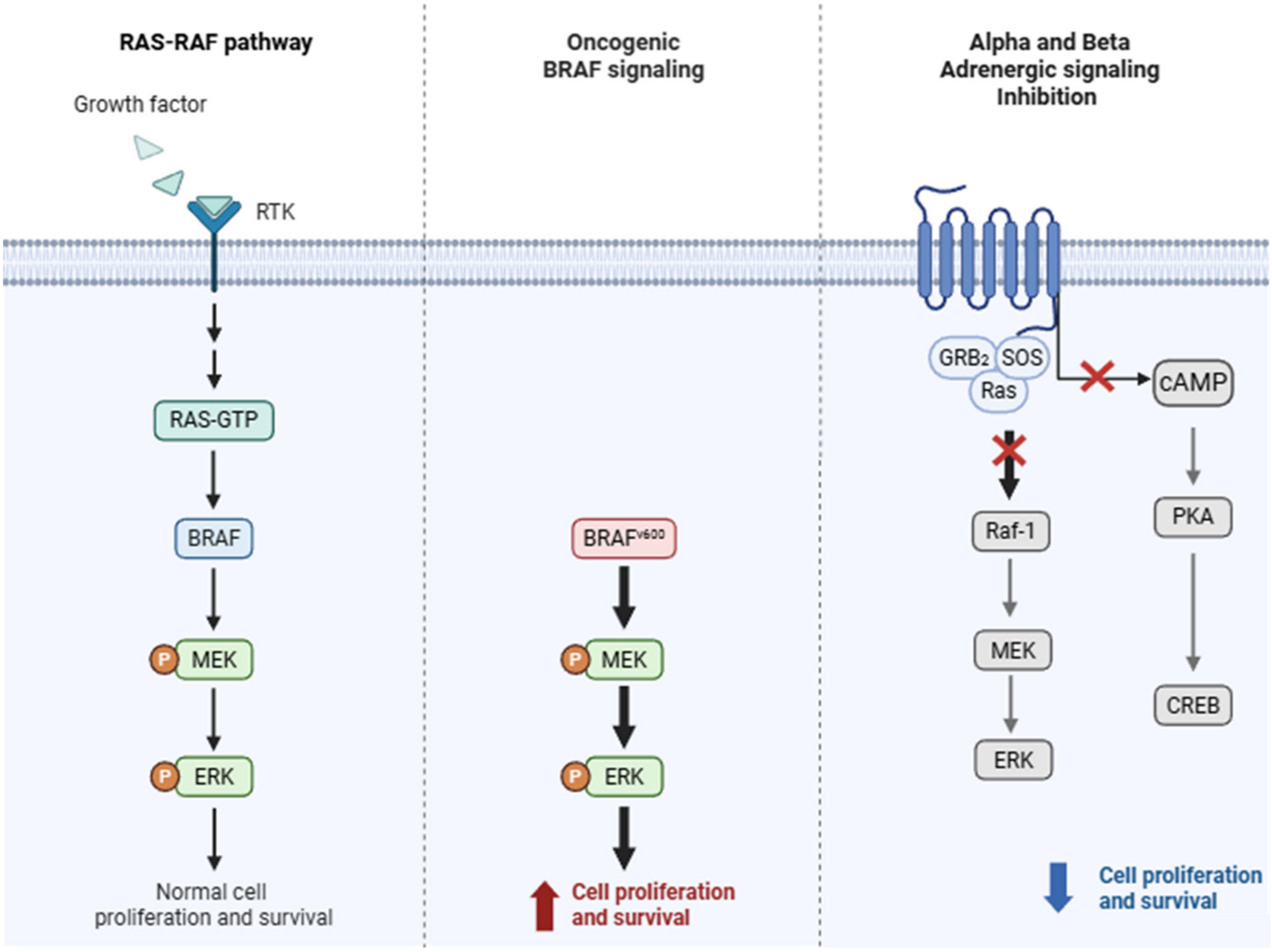

Figure S3: β-adrenergic signaling crosstalk with BRAF V600E mutation in melanoma.
